# Surgical Margins in Breast Conservation

**DOI:** 10.1155/2013/136387

**Published:** 2013-01-21

**Authors:** Sheldon Marc Feldman

**Affiliations:** Division of Breast Surgery, Vivian L. Milstein Associate Professor of Clinical Surgery, College of Physicians and Surgeons, Columbia University, 161 Fort Washington Avenue, 10th Floor, Suite 1005, NY 10032, USA

Significant progress has been made in the diagnosis and treatment of breast cancer during the past 30 years. The increased availability of screening mammography has resulted in a higher percentage of woman being diagnosed with early stage disease allowing the option of breast conservation therapy to be more widely available. Long-term follow-up studies clearly demonstrate equivalent survival with breast conservation surgery (lumpectomy) and radiotherapy versus total mastectomy [1–3]. The importance of obtaining clear lumpectomy surgical margins has been well established in minimizing the risk of local recurrence [4]. Unfortunately there is a lack of uniform guidelines in terms of what constitutes an adequately clear lumpectomy margin. Substantial debate about bigger margins being better continues [5]. This has led to wide variations in lumpectomy margin reexcision rates from 15 to 47% [6]. These additional surgical procedures cause significant patient distress, utilize health care resources, and can adversely affect cosmesis. From the patient perspective, they may wonder why we did not get it right the first time. They want their cancer gone while maintaining a normal appearance.

This special issue highlights the areas of controversy and demonstrates current best practices and emerging novel approaches towards optimal breast conservation approach. The goal is to improve our ability to provide breast-conserving approaches for breast cancer while avoiding multiple surgical procedures, minimizing recurrence risk while obtaining excellent cosmesis. We have chosen 6 of 16 submissions to be published in this special issue. Each paper was evaluated by at least two expert reviewers and revised according to review comments.

P. Ananthakrishnan et al. provide an excellent comprehensive review article on all aspects involved in optimizing breast conservation. They include discussion of preoperative breast imaging, lesion localization, impact of tumor biology and systemic therapy, intraoperative lesion identification and margin assessment techniques, the role of margin ablation and oncoplastic techniques. They also discuss the promise of ductal anatomy mapping toward the goal of validating the “Sick lobe hypothesis” [7, 8] which may allow for more accurate identification of breast tissue to be targeted for excision.

R. Emmadi and E. L. Wiley provide an excellent review from the pathology perspective of the different approaches to margin assessment. They explore issues of specimen processing, fixation, cutting techniques, and reporting. They well explain the reasons for the reporting variations between institutions and the need for standardization.

J. L. Baker et al. present a scholarly review of our current understanding of the issue of atypical ductal hyperplasia (ADH) as it relates to surgical margins. They highlight the large interobserver variability among pathologists in differentiating ADH from low-grade ductal carcinoma in situ (DCIS). The issue of whether ADH is a precursor lesion to DCIS is explored. 

R. J. Rivera et al. report on a 21-site multicenter clinical trial evaluating the performance of the MarginProbe intraoperative device. This device is based on radiofrequency spectroscopy to assess adequacy of lumpectomy margins. They analyzed volume or resection and reexcision rates in the device group versus usual surgical standard of care (SOC). They demonstrate the reexcision rate of 14.1% in the device group versus 29.9% with SOC. Increased resection volume was 2.6% using the device.

M. M. Chang et al. provide a comprehensive overview of oncoplastic breast reduction. This is a complete review of the techniques including indication, patient selection, practical pointers, and their experience including a low (3.3%) rate of margin failure. They stress the importance of a coordinated team approach between breast surgical oncology, plastic surgery, breast imaging, and radiation oncology.

Lastly, G. H. T. Au et al. present an exciting research paper on margin assessment using a Quantum-Dot Molecular probe in a mouse model. This employs nanoparticle monoclonal antibodies with molecular imaging. Their concept has a potential advantage over optical imaging and radiofrequency spectroscopy in that it is not affected by tissue heterogeneity. It also can display and differentiate very small (100–200 cells) spots. Timeline of 30 minutes is practical for intraoperative use. This early work is an highly innovative approach to a practical issue.

These papers present a great deal of important information and well explore the current state of the art, controversies and future directions towards the important goal of optimizing breast conservation with particular attention to margin issues. 
